# Angiotensin II Regulates microRNA-132/-212 in Hypertensive Rats and Humans

**DOI:** 10.3390/ijms140611190

**Published:** 2013-05-27

**Authors:** Tilde V. Eskildsen, Pia L. Jeppesen, Mikael Schneider, Anne Y. Nossent, Maria B. Sandberg, Pernille B. L. Hansen, Charlotte H. Jensen, Maria L. Hansen, Niels Marcussen, Lars M. Rasmussen, Peter Bie, Ditte C. Andersen, Søren P. Sheikh

**Affiliations:** 1 Department of Cardiovascular and Renal Research, Institute of Molecular Medicine, University of Southern Denmark, DK-5000 Odense, Denmark; E-Mails: teskildsen@health.sdu.dk (T.V.E.); piajeppesens@hotmail.com (P.L.J.); a.y.nossent@lumc.nl (A.Y.N.); plaerkegaard@health.sdu.dk (P.B.L.H.); charken@health.sdu.dk (C.H.J.); pbie@health.sdu.dk (P.B.); dandersen@health.sdu.dk (D.C.A.); 2 Department of Clinical Biochemistry and Pharmacology, Odense University Hospital, Sdr. Boulevard 29, DK-5000 Odense, Denmark; E-Mails: mikaelschneider@gmail.com (M.S.); maria.sandberg@ouh.regionsyddanmark.dk (M.B.S.); mlhansen@health.sdu.dk (M.L.H.); lars.melholt.rasmussen@ouh.regionsyddanmark.dk (L.M.R.); 3 Department of Vascular Surgery, Einthoven Laboratory for Experimental Vascular Medicine, Leiden University Medical Center, 2333 ZA Leiden, The Netherlands; 4 Department of Cardiac and Thoracic Surgery, Odense University Hospital, Sdr. Boulevard 29, DK-5000 Odense, Denmark; 5 Department of Pathology, University of Southern Denmark, DK-5000 Odense, Denmark; E-Mail: nmarcussen@health.sdu.dk

**Keywords:** hypertension, Angiotensin II, AT_1_R, AT_1_ receptor blocker, microRNA

## Abstract

MicroRNAs (miRNAs), a group of small non-coding RNAs that fine tune translation of multiple target mRNAs, are emerging as key regulators in cardiovascular development and disease. MiRNAs are involved in cardiac hypertrophy, heart failure and remodeling following cardiac infarction; however, miRNAs involved in hypertension have not been thoroughly investigated. We have recently reported that specific miRNAs play an integral role in Angiotensin II receptor (AT_1_R) signaling, especially after activation of the Gαq signaling pathway. Since AT_1_R blockers are widely used to treat hypertension, we undertook a detailed analysis of potential miRNAs involved in Angiotensin II (AngII) mediated hypertension in rats and hypertensive patients, using miRNA microarray and qPCR analysis. The miR-132 and miR-212 are highly increased in the heart, aortic wall and kidney of rats with hypertension (159 ± 12 mm Hg) and cardiac hypertrophy following chronic AngII infusion. In addition, activation of the endothelin receptor, another Gαq coupled receptor, also increased miR-132 and miR-212. We sought to extend these observations using human samples by reasoning that AT_1_R blockers may decrease miR-132 and miR-212. We analyzed tissue samples of mammary artery obtained from surplus arterial tissue after coronary bypass operations. Indeed, we found a decrease in expression levels of miR-132 and miR-212 in human arteries from bypass-operated patients treated with AT_1_R blockers, whereas treatment with β-blockers had no effect. Taken together, these data suggest that miR-132 and miR-212 are involved in AngII induced hypertension, providing a new perspective in hypertensive disease mechanisms.

## 1. Introduction

Persistent elevation of systemic blood pressure (hypertension) is one of the most prevalent medical conditions involving the cardiovascular system and affects as many as one billion people worldwide [1]. Hypertension is an undisputed risk factor for cardiovascular diseases, including stroke, cardiac failure and renal diseases [1]. Several mechanisms have been implicated in the pathogenesis of hypertension, including increased activity of the sympathetic nervous system, dysfunction of the vascular endothelium, vascular smooth muscle and cardiac hypertrophy, as well as overactivation of the renin-angiotensin-aldosterone system (RAAS) [2]. Angiotensin II (AngII) controls blood pressure and fluid homeostasis through its receptors, AT_1_R and AT_2_R, and through stimulation of aldosterone [1]. AngII receptors are expressed in tissues that have an impact on blood pressure control, including heart, kidney and vasculature [3,4]. The classical AngII responses in the cardiovascular and renal systems are mediated mainly by AT_1_R signaling [3–5], including heterotrimeric G-protein activation and downstream signaling through the canonical MAP kinases ERK1/2, which, in turn, regulate gene transcription [4]. Accordingly, specific inhibitors of AngII pathways, including AT_1_R blockers, dramatically lower blood pressure in hypertensive patients and slow the progression of cardiovascular disease [1,3].

We speculated that altered expression of microRNAs (miRNA) may be part of the pathogenesis behind AngII-related hypertension. MicroRNAs are small non-coding RNAs that regulate gene expression by pairing to and destabilizing the mRNAs of protein coding genes, resulting in decreased mRNA levels [6]. The mammalian miRNAs are highly conserved, and each miRNA is predicted to target mRNAs of hundreds of distinct genes, fine-tuning and optimizing the expression patterns of most protein-coding genes [7]. Theoretically, these miRNAs are ideally suited to co-regulate gene expression events in cellular responses to vasopressors, such as AngII. Most miRNAs are solitary and expressed under the control of their own promoters and regulatory sequences, while others are arranged as clusters and may be co-regulated with additional members of the cluster [7]. For example, miR-132 and miR-212 are clustered closely in the genome and are transcribed together under the regulation of cAMP response element binding protein [8], which is a known AngII regulated gene [9,10]. Several miRNAs are aberrantly expressed in cardiovascular diseases [2,11–13]. miR-21, miR-155 and miR-221/222 have recently been shown to regulate AngII signaling in cardiac fibroblasts [14–16] and in endothelial cells [17], while miR-29 regulates fibrotic pathways [18]. We have recently shown that AngII, via the Gαq pathway, regulates five miRNAs during *in vitro* stimulation of primary cardiac fibroblasts and of HEK293N cells overexpressing the AT_1_-receptor [19]. Most of the miRNA studies are based on *in vitro* experiments, and very few studies have examined the relation between AngII mediated hypertension and miRNA regulation *in vivo*.

In this study, we hypothesized that *in vivo* AngII mimics the “five miRNA” expression signature obtained by AT_1_R overexpression [19]. We examined the miRNA expression in heart, aorta and kidney from a rat model with a constant intravenous infusion of AngII and expanded these results to data concerning miRNA expression in the mammary artery of patients treated with AT_1_R blockers. Our results suggest that miR-132 and miR-212 are involved in AngII-induced Gαq-signaling pathway leading to hypertension. Further understanding of the importance of these miRNAs will come from future miRNA knockdown experiments or knockout in whole animals.

## 2. Results

### 2.1. High Blood Pressure, Cardiac Hypertrophy and Fibrosis Are Sustained in the Rat Model

**Figure 1:**
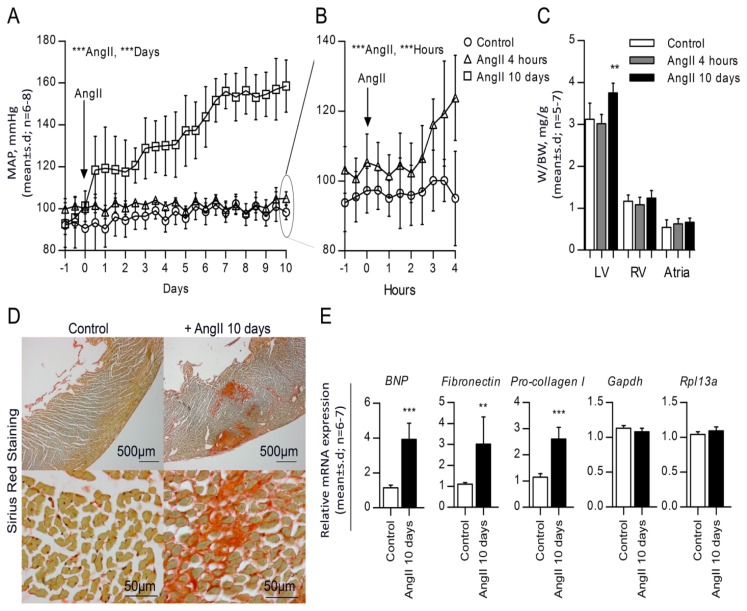
Characterization of AngII-induced hypertensive rat. (**A**) Mean daily averages of mean arterial blood pressure from seven rats treated with chronic infusion of 30 ng/kg/min AngII for 10 days (□) and six rats treated with acute infusion of 30 ng/kg/min AngII for 4 h (Δ), compared to eight control rats (○); (**B**) Mean hourly averages of mean arterial blood pressure from acute infusion of rats (Δ) for four h compared to control rats (○). Data are shown as the mean ± SD. Arrows shows the start of AngII infusion (day 0). Statistical significance was tested by two-way ANOVA for either control *versus* AngII for 10 days or control *versus* AngII for 4 h. ***p < 0.001. A and B, duplicate figure [20]; (**C**) Weight to body weight ratio (mg/g) of chronic (*n =* 7), acute (*n =* 6) and control (*n =* 8) rat hearts divided into left ventricle, right ventricle and atria. Data is presented as the mean ± SD, and statistical significance was tested by one-way ANOVA using Tukey’s multiple comparison test. **p < 0.01; (**D**) Representative sections of left ventricles of AngII affected hearts compared to control hearts, stained with Sirius Red for collagen deposition; (**E**) qRT-PCR for the early marker of hypertrophy, *BNP,* the fibrotic markers, *Fibronectin* and *Procollagen-I*, and the two stably expressed reference genes, *Gapdh and Rpl13a* (M: 0.140 and CV: 0.049). Data is presented as the mean ± SD, and statistical significance was tested by un-paired *t*-test. *p < 0.05, **p < 0.01 and ***p < 0.001.

Infusion of AngII for 10 days resulted in a stable and significant elevation in blood pressure to 159 ± 12 mm Hg (*p* < 0.001, *n =* 7) at day 10, as compared to control rats that remained constant at 98 ± 4 mm Hg (*n =* 8) (Figure 1A). Likewise, we found that short time (4 h) AngII infusion resulted in an acute and significant 29 mm Hg increase in blood pressure (*p* < 0.001, *n =* 6) (Figure 1B). AngII hypertensive rats exhibited cardiac hypertrophy, as evidenced by a significant 17% increased left ventricle to body weight ratio (*p* < 0.01, *n =* 7) *versus* control rats (*n =* 6) (Figure 1C). The mass of the left ventricle increased from 614 ± 82 mg (*n =* 6) in control rats to 780 ± 75 mg (*n =* 7) in the hypertensive rats (*p* < 0.01), whereas no increase was observed for the right ventricle or atria weight (Figure 1C). Left ventricular hypertrophy was further validated by a significantly higher expression level of *B-type natriuretic peptide* (*BNP*) (*p* < 0.001) (Figure 1E). Likewise, cardiac fibrosis after infusion of AngII for 10 days was confirmed by increased collagen deposition (Figure 1D) and an increased expression of genes generally associated with fibrosis, including *Fibronectin* (*p* < 0.01) and *Procollagen I* (*p* < 0.001) (Figure 1E). These results thus showed that continuous AngII infusion for 10 days resulted in clear and sustained hypertension, leading to hypertrophic and fibrotic changes of the heart.

### 2.2. Chronic AngII-Mediated Hypertension in Rats Increases miR-132/-212 Cluster Expression in Blood Pressure Regulating Organs: Heart, Aorta and Kidney

**Figure 2:**
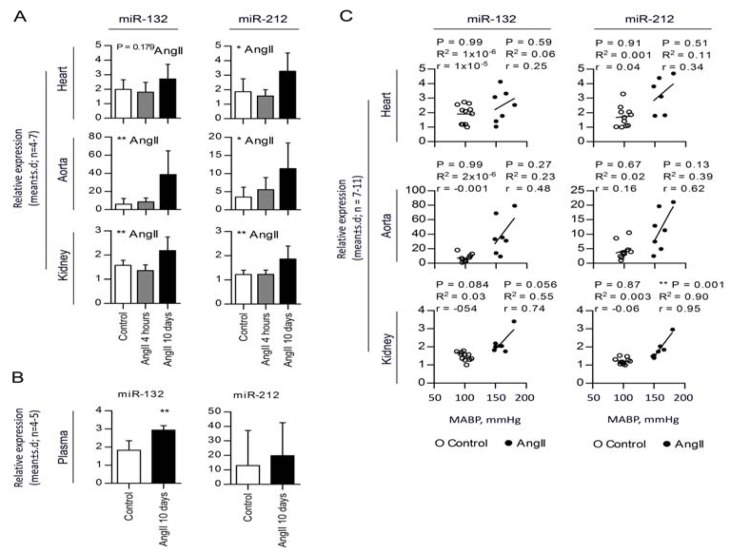
Validation of miRNA regulation in AngII-induced hypertensive rat heart, aorta and kidney. (**A**) qRT-PCR identification of miRNAs in left ventricle of hearts, aortas and kidneys from AngII affected rat hearts. Statistical significance was tested by one way ANOVA. *p < 0.05. Values are shown as relative expression with the mean ± SD; *n =* 4–7; (**B**) qRT-PCR identification of miRNAs in plasma of AngII affected rat hearts. Statistical significance was tested by un-paired *t*-test. **p < 0.01. Values are shown as relative expression with the mean ± SD; *n =* 4–5. miRNA expression in the three organs and in plasma was individually normalized to two reference genes stably expressed among the samples. Reference genes used for normalization: Heart, miR-17 and miR-191 (M: 0.356, CV: 0.123); aorta, miR-103 and miR-191 (M: 0.832, CV: 0.290); kidney, miR-17 and miR-191 (M: 0.145, CV: 0.050); and plasma, miR-17 and miR-103 (M: 1.143, CV: 0.390); (**C**) Correlation analysis for miRNA expression levels at day 10 of AngII or isotone glucose infusion. Statistical significance was tested by linear regression (*R*^2^) and correlation analysis (Pearson’s *r*). Data are shown as two individual correlations per miRNA is each organ for control (○) and AngII for 10 days (●). **p < 0.01; *n =* 7–11.

Fifty miRNAs were identified as differentially expressed (*p* < 0.05) in hearts of sustained hypertension (10 days of AngII), as compared to controls (Table 1), whereas no miRNAs were differentially expressed in acute hypertensive rats (4 h of AngII). AngII affects the blood pressure by two separate mechanisms: firstly, an acute contractile effect on the arterial walls arising within a few hours, followed by a chronic compensative response arising after a few days. Gene expression is primarily effected by the long-term AngII signaling. However, we sought to investigate if miRNAs could rapidly change, due to the acute effect, and found no regulation of miRNAs in the short period of AngII infusion. Of the 50 miRNAs differentially regulated in chronic hypertension, miR-21 was the most upregulated and was used as a positive control in our analysis. Interestingly, among the many dysregulated miRNAs, the second most significantly regulated miRNA was miR-132. Since the miR-132 gene is clustered with the miR-212 gene and they are likely expressed together [21], we included miR-212 in further analyses. In summary, we found that the expression of miR-21, -132 and -212 were up-regulated by 3.4-, 1.4- and 1.8-fold, respectively, in the left ventricle of AngII-induced hypertensive animals, as compared to controls (Figures 2A and S1). Besides the heart, the arterial wall and kidneys are involved in systemic blood pressure homeostasis, and we, therefore, examined whether the miR-132/-212 levels were affected also in these tissues. miR-132 and -212 were increased 6.4- and 3.2-fold, respectively, in aortas from hypertensive animals, as compared to controls (Figure 2A). Likewise, we observed a significant regulation of miR-132 (1.4-fold) and -212 (1.5-fold) in the kidneys of AngII-infused rats *versus* controls (Figure 2A). Furthermore, miR-132 was found to be significantly upregulated in the plasma of AngII-induced hypertensive animals, whereas no regulation was observed for plasma miR-212 levels compared to the control rats (Figure 2B). Lack of miR-212 upregulation in the plasma of AngII-induced hypertensive rats compared to control rats might be caused by the low concentration and high variability of the miR-212 levels found in the plasma. Of note, the miRNA expression levels were found to significantly correlate with AngII-induced hypertension in the kidneys, a strong tendency of correlation in aorta and a lower tendency for correlation in the heart as tested by linear regression and correlation analysis (Figure 2C). By contrast, no changes in miR-132/-212 levels were found in any of the three tissues in the acutely hypertensive rats (Figure 2A). These data confirmed the microRNA array data obtained for chronic and acute hypertensive rats. Altogether, these results strongly suggest that the miR-132/-212 cluster may be a general and novel mediator of AngII-induced hypertension.

### 2.3. miR-132 and -212 Regulation in Response to AngII in Mice

**Figure 3:**
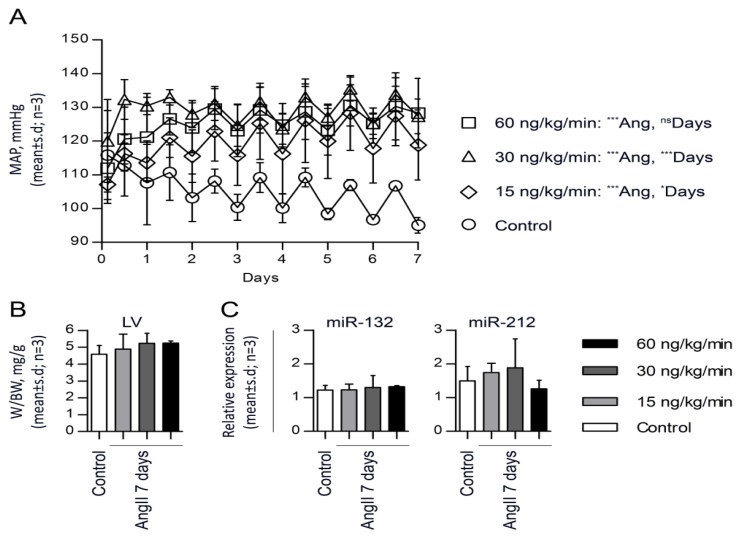
Validation of miRNA regulation in AngII-induced hypertensive mice hearts. (**A**) Mean daily averages of mean arterial blood pressure from three mice treated with chronic infusion of 60, 30 or 15 ng/kg/min AngII for seven days (□, Δ or ◇, respectively), compared to three control mice (○). Data are shown as the mean ± SD. AngII infusion is started at day 0. Statistical significance was tested by two-way ANOVA. ***p < 0.001, *p < 0.05; (**B**) Left ventricle (LV) weight to body weight ratio (mg/g) of mice infused with 60, 30 or 15 ng/kg/min of AngII for seven days (*n =* 3) and control (*n =* 3) mice. Data is presented as the mean ± SD, and statistical significance was tested by one-way ANOVA using Tukey’s multiple comparison test; (**C**) qRT-PCR identification of miRNAs in left ventricle of hearts from AngII affected mice hearts. Statistical significance was tested by one-way ANOVA using Tukey’s multiple comparison test. Values are shown as relative expression with the mean ± SD; *n* = 3. Reference genes used for normalization: miR-103 and miR-191 (M: 0.826, CV: 0.288).

Studying mice in this particular field of hypertension research is of interest, because their genome can easily be modified. Infusion of AngII at 15, 30 and 60 ng/kg/min for seven days resulted in a stable and significant elevation in blood pressure to 119 ± 10, 127 ± 5 and 128 ± 10 mm Hg (*p* < 0.001, *n =* 3), respectively, at day seven, as compared to control mice that remained constant at 95 ± 2 mm Hg (*n =* 4) (Figure 3A). AngII-infused mice did not exhibit cardiac hypertrophy when examining the left ventricle to body weight ratio (*n =* 3) *versus* control mice (*n =* 4) (Figure 3B). Likewise, miR-132 and miR-212 were not significantly increased in AngII-infused mice at any of the AngII concentrations when compared to control mice. These results thus showed that continuous AngII infusion for seven days in mice resulted in a modest increase in blood pressure without hypertrophic changes of the heart and no regulation of the miR-132/-212 cluster.

### 2.4. miR-132 and -212 Regulations in Response to ET-1, Vasopressor-Induced Hypertension

**Figure 4:**
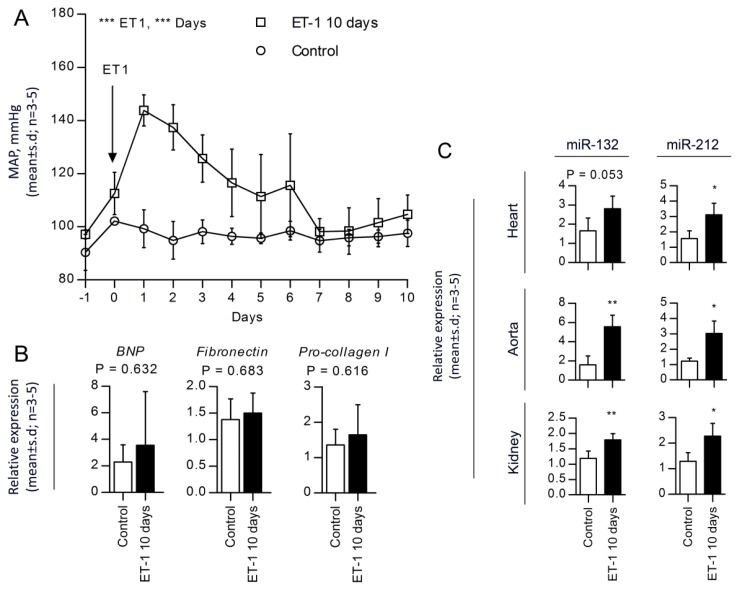
Endothelin 1-induced hypertensive rats. (**A**) Mean daily averages of mean arterial blood pressure from five rats treated with chronic infusion of 5 pmol/kg/min ET-1 for 10 days (□), compared to three control rats (○). Statistical significance was tested by two-way ANOVA. ***p < 0.001; (**B**) qRT-PCR for the early hypertrophy marker, *BNP* and the fibrosis markers, *Fibronectin* and *Procollagen-I*. Statistical significance was tested by un-paired *t*-test. Data is shown as the mean ± SD, *n =* 3–5. Reference genes used for normalization: *GAPDH* and *Rpl13a* (M: 0.153, CV: 0.053); (**C**) qRT-PCR identification of miRNAs in the left ventricle of hearts, aortas and kidneys from ET-1 affected rat hearts. Statistical significance was tested by un-paired *t*-test. *p < 0.05. Values are shown as relative expression with the mean ± SD, *n =* 3–5. miRNA expression was individually normalized to two reference genes stably expressed among the samples. Reference genes used for normalization: heart; miR-17 and miR-191 (M: 0.309, CV: 0.107); aorta, miR-103 and miR-191 (M: 0.667, CV: 0.232); kidney, miR-17 and miR-191 (M: 0.180, CV: 0.062).

In order to test whether the miR-132/-212 cluster response is specific for AT_1_R induction, we examined the effect of continuous infusion of a second vasopressor (ET-1) [22]. In contrast to the sustained high blood pressure in AngII-infused rats, ET-1-mediated hypertension peaked at day one (144 ± 6 mm Hg (*n =* 5) and, hereafter, gradually declined, reaching 105 ± 7 mm Hg at day 10 (Figure 4A). In line with this, we did not observe any cardiac hypertrophy or fibrosis in ET-1 treated hearts. Thus, we found no change in *BNP*, *Fibronectin* or *Procollagen-I* expression levels (Figure 4B). However, despite the partially compensated blood pressure and the absence of cardiac hypertrophy and fibrosis, we found significantly higher levels of miR-132 and miR-212 in heart (1.7- and 2.0-fold), aorta (3.5- and 2.5-fold) and kidney (1.5- and 1.8-fold) of the ET-1 treated animals as compared to controls (Figure 4C). In line with the previous findings by Jeppesen *et al.* [19], this suggests that the upregulation of miR-132/-212 is a direct consequence of Gαq vasopressor stimulation pathways and not a result of secondary heart hypertrophy and fibrosis. In summary, these data indicate that upregulation of the miR-132/-212 cluster likely is part of a general response to Gαq-vasopressor stimulation of the ERK1/2 pathway and may be involved in a common AngII- and ET-1-mediated signaling pathway leading to hypertension.

### 2.5. Treatment with Angiotensin II Receptor Blocker Attenuates the Expression of the miR-132/-212 Cluster in Human Hypertension

**Figure 5:**
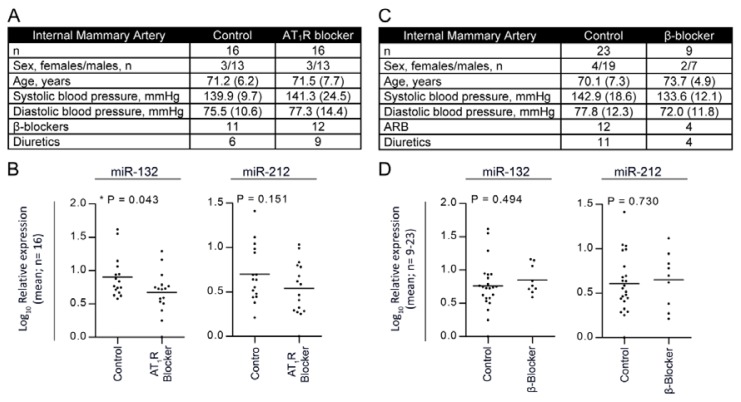
AngII receptor blockers and β-blockers in internal mammary artery (IMA) patients. (**A**,**C**) Study group information on the AngII receptor blocker (ARB) and β-blocker patient groups, respectively. None of the characteristics within the patient groups of ARBs and β-blockers were significantly different. The groups were matched for age, sex, diabetes and treatment using statins. None of the patients were simultaneously treated with angiotensin-converting enzyme (ACE)-inhibitors; (**B**) Scatter plot represents Log_10_ relative miRNA expression with mean bar, in patients treated with ARBs compared to patients treated with non-ARB (*n =* 16). (**D**) Scatter plot representing Log_10_ relative miRNA expression with mean bar, in patients treated with β-blocker (*n =* 9) compared to patients treated with non-β-blockers (*n =* 23). Expression is normalized to two stably expressed reference genes. Reference genes used for normalization: miR-103 and miR-191 (M: 0.804, CV: 0.279).

We measured miR-132/-212 levels in the internal mammary artery (IMA) tissue obtained from patients treated with AngII receptor blocker (ARB) or β-blocker in age and sex matched patients undergoing by-pass surgery (Figure 5A,C). The four patient groups showed similar pre-operative blood pressure (Figure 5A,C). ARB treated patients (*n =* 16) revealed a significant attenuation of miR-132 expression (0.55-fold), as well as a tendency for miR-212 downregulation (0.64-fold), as compared to non-ARB-treated patients (miR-132; 0.93 and miR-212; 1.01) (*n =* 16) (Figure 5B). To further investigate whether this miR-132 and -212 regulation is specific to AngII or related to putative direct influences from blood pressure, we examined patients treated with β-blockers (Figure 5C). Interestingly, we did not find any attenuation or downregulation of miR-132/-212 expression in IMA patients receiving β-blockers, as compared to non-β-blocker-treated patients (Figure 5D), indicating that not all blood pressure-reducing agents can downregulate miR-132/-212 expression, which further supports the notion that AngII mediates a global upregulation of miR-132/-212 in humans. These results suggest that the miR-132/-212 cluster in humans may also be part of the response to Gαq-vasopressors, such as AngII.

## 3. Discussion

We found increased expression of miR-132 and -212 in the left ventricle, aorta and kidney, as well as in the plasma (Figure 2) after 10 days of sustained AngII-induced hypertension in rats, which is compatible with our pervious published *in vitro* study [19]. Even though miR-132 and miR-212 are expressed from the same precursor, we observed independent regulation in the different tissues in response to the same AngII infusion. This could be due to differences in stability and degradation [21,23]. Moreover, the degree of miR-132/-212 increase shows a tendency to correlate with blood pressure suggesting that these miRNAs could play a novel role in AT1 receptor pharmacology, both *in vitro* and *in vivo*.

Blood pressure has previously been associated with miRNA. Recently, Nossent *et al.* investigated the association between single-nucleotide polymorphisms (SNPs) located in the miRNA binding sites of genes associated with Renin-AngiotensinII-system (RAS), blood pressure and myocardial infarction in a large population study [24]. Several SNPs located in RAS genes were associated with changes in blood pressure and were shown to interfere with miRNA regulation [24].

miR-132/-212 has been described in both the central nervous and cardiovascular systems. In one study, miR-132 was reported to be constitutively expressed and released by pericyte progenitor cells, and transplantation of these cells into mice with myocardial infarction improves cardiac function through proangiogenic activities [25]. Several studies identify miR-132/-212 involvement in the central nervous system, *i.e.*, in neuronal function and plasticity [8,21,26]. In addition, miR-132/-212 has also been found to be involved in neovascularization, inflammation and adipocyte differentiation in the peripheral tissues [21,23,27]. Interestingly, miR-132/-212 was upregulated in the aortas of mice stimulated with AngII from osmotic pumps for 14 days, but this study did not report blood pressure values [28]. We performed similar studies with chronic AngII infusion in mice for seven days, simultaneously measuring blood pressure. In contrast to our rat infusion model, only a modest blood pressure increase was observed in this mice strain, and miR-132/-212 was not significantly upregulated in mouse hearts (Figure 3). Differences in AngII effects between rats and mice have previously been described by Cassis *et al.*, stating that AngII infusion in mice has minimal effects compared to same doses given to rats [29]. Based on these data, we decided that the rat model was more suitable for studying AngII-induced miRNA changes in hypertensive animals. We previously demonstrated that AT_1_R signaling regulates miR-132 and -212 in HEK293N cells and in primary cultures of cardiac fibroblasts through the Gαq dependent pathway [19]. These results were recently confirmed in primary cultures of rat vascular smooth muscle cells [28]. Additionally, we investigated the regulation of the miR-132/-212 cluster in endothelial cell lines and in primary cultures of vascular smooth muscle cells (VSMCs) and leukocytes and found no regulation in either of the cell types (data not shown). Furthermore, by inhibition of the Gαq subunit in cardiac fibroblasts, we demonstrated significant decreases in miR-132 and -212 expressions, pointing to Gαq protein activation as the responsible pathway for AngII-induced miRNA regulation *in vitro* [19].

Using a different vasopressor (ET-1) that binds to the endothelin receptor and also activates Gαq-ERK1/2 signaling [30,31], we examined whether this receptor could regulate the miRNAs. Interestingly, even though the ET-1-induced hypertension had a much shorter duration than the sustained hypertension induced by AngII, both miR-132 and miR-212 were upregulated at a point in time when blood pressure was not (Figure 4). From a mechanistic point of view, these findings indicate that miR-132/-212, also *in vivo*, may be regulated through Gαq-ERK1/2 activation, which is one of the mutual steps in the AngII and ET-1 signaling pathways leading to hypertension [30–32]. Following these observations, we examined whether the increased miRNA expression levels could be attenuated by blocking the AngII signaling in humans (Figure 5). We examined the arterial walls from two different groups of patients treated with either one of several ARBs, including Losartan, Candesartan, Irbesartan and Telmisartan, or no ARB. These ARBs are widely used in hypertension treatment and have similar chemical structures, but different pharmacological properties and efficacy [33]. None of the patients were treated with ACE-inhibitors. Our patient groups were selected based on treatment with ARBs and individual ARB-types grouped and analyzed together in order to allow for statistical testing. Most patients in the ARB group probably suffered from hypertension; however, it has not been possible from the patient files to deduce who strictly fulfills the definitions of hypertension. Likewise, in the non-ARB group, the precise prevalence of hypertension is not known. Another limitation of our human observations is the lack of knowledge about the exact time point of medication, which could not be controlled. Despite these limitations, we observed a significant downregulation of miR-132, as well as a robust attenuation of miR-212 in the ARB-treated patients. We next asked whether treatment with β-blockers, often a first choice antihypertensive drug, would also decrease miR-132 and -212, following the notion that it could be reduction in blood pressure *per se*, which may alter the miRNA levels. However, the levels of miR-132/-212 were not downregulated in patients treated with β-blockers. This observation is compatible with the notion that alterations in miRNA is not only a spurious factor found in rodents or related to experimental systems *in vitro*, but that these molecules also could be suggested to play a role in the arterial wall among patients receiving medication that alters the activity in the AngII system. Our data indicates that AT_1_R control of these miRNAs is evolutionary conserved between rat and man. Thus, AT_1_R activation in rats increases miR-132 and miR-212, while blocking the AT_1_R decreases miRNA levels in humans. Since no anti-miR experiments have been conducted, it has not been possible to deduce the specific cause and effect relationship; however, the regulation of miR-132 and miR-212 is likely biological important, because although rats and humans share biological features in blood pressure control, they have multiple differences in the molecular subtypes of ion channels, receptors and signaling pathways in blood vessel cells. Further studies are necessary to assess the relative biological and pharmacological impact of individual miR-132 and miR-212 levels on systemic blood pressure, in the heart, arterial wall and kidney. We speculate that the AT_1_R could perform these effects through Gαq and downstream activation of the ERK1/2 pathway and not through blood pressure or aldosterone. Three lines of evidence support this notion. Firstly, the effect was inhibited by the pharmacological blockade of AngII *in vivo* and Gαq and ERK1/2 *in vitro* [19]. Secondly, another Gαq activator (ET-1) reproduced the effects, while, finally, inhibiting Gs signaling with the β-adrenergic blocker had no effects on the miRNA levels.

## 4. Experimental Section

### 4.1. Animal Care

Female Sprague-Dawley rats (8 weeks old, Taconic, Ry, Denmark) were housed in air-conditioned rooms with a 12 h dark-light cycle and fed standard diet (Altromin^®^ Standard 1320, Lage, Germany) with free access to tap water. All rat experiments were approved by the Danish National Animal Experiment Inspectorate (Permission #2009/561/1753).

### 4.2. Angiotensin II (AngII) Model

Under Hypnorm/midazolam anesthesia, chronic catheters were placed in the left femoral vein and artery and connected to a swivel via a skin button between the scapulae, allowing the rat full mobility [34]. Following a 5–7 day recovery period, the arterial line was connected to a pressure transducer (Föhr Medical Instruments, Hessen, Germany), and data were collected continuously using Lab View software (National Instruments, Austin, TX, USA). The rats were infused with 5% glucose solution at a rate of 10 μL/kg/min with or without AngII (Sigma-Aldrich, St. Louis, MO, USA) for 10 days. Rats were randomly divided into three groups: (1) 10 days’ continuous infusion of AngII at 30 ng/kg/min (sustained hypertension); (2) 9 days’ and 20 h infusion with 5% glucose, followed by a 4 h period of AngII infusion at 30 ng/kg/min (acute hypertension); and (3) 10 days’ infusion of 5% glucose (controls). Rats were sacrificed with an overdose of pentobarbital, then perfused with ice-cold sterile isotonic saline via the heart. The heart, kidney and aorta were dissected under sterile conditions. Hearts were carefully sectioned into atria, right ventricle and left ventricle, whereas aortas were cleaned from fat, as well as connective tissue, and kidneys were decapsulated.

### 4.3. Endothelin 1 (ET-1) Model

The setup was similar to that described above for the AngII model, but rats were randomly divided into two groups: (1) 10 days’ infusion of endothelin-1 (ET-1) (Bachem) at a concentration of 5.0 pmol/kg/min in isotonic saline; and (2) 10 days’ infusion with isotonic saline, as previously described by Mortensen L.H. & Fink G.D. 1992 [35].

### 4.4. Mice AngII Model

The mice experiments were approved by the Danish National Animal Experiment Inspectorate (Permission #2009/561/1749). The setup was similar to that described above for the AngII rat model with minor modifications as described below. Mixed gender C57/BL6 mice were anaesthetized using ketamine/xylazine and catheters placed in the femoral artery and vein for measurements of arterial blood pressure and AngII infusions, respectively. The mice recovered for five days before beginning the continuous measurements of mean arterial blood pressure and heart rate for seven days. Mice were randomly divided into four groups: (1–3) 7 days’ continuous infusion of AngII at 15, 30 and 60 ng/kg/min respectively and (4) 7 days’ of continuous infusion with 5% glucose (controls). Mice were sacrificed and processed, as described above.

### 4.5. Patients, Internal Mammary Artery Study

Surplus arterial tissue was obtained from the repair vessel, *i.e.*, the internal mammary artery (IMA), from patients undergoing coronary artery by-pass graft surgery at the Department of Cardiac and Thoracic Surgery, Odense University Hospital, Denmark. Four groups of patients were analyzed, based on intake or no intake of either AngII receptor blocker or β-blocker. Study population characteristics are described in (Figure 5A,C). As previously described [36], the intima/media of the artery was carefully dissected immediately after removal, frozen in liquid nitrogen and stored in the Odense Artery Biobank. The study was approved by the Ethical Review Board of Southern Denmark (No. S-20100044), and informed consent was obtained from each subject.

### 4.6. Sirius Red Staining

Fibrosis was identified by Sirius Red Staining for collagen deposits. Cryosections were equilibrated at room temperature for 30 min, rinsed in a mixture of NBF (37%) and ethanol (93%) for 45 s, rehydrated (5 min) and subsequently counterstained (15 min) with Weigert’s Iron Hematoxylin (Sigma-Aldrich, HT-107/109). Following a wash, sections were further incubated (60 min) with 0.1% Sirius red (Sigma-Aldrich, P6744) in saturated picric acid (Sigma-Aldrich, 365548) and finally washed twice in 99% ethanol. Sections were mounted with Pertex and analyzed by bright-field microscopy using a Leica DML332 equipped with a Leica DFC300F camera.

### 4.7. Microarray and Data Processing

The miRNA expression profiling was performed as two-color common reference hybridizations on LNA-based arrays (miRCURY LNA™ microRNA Array ready to spot probe set, Exiqon, Denmark) spotted in-house on CodeLink™ HD Activated slides (DHD1-0023, SurModics, Eden Prairie, MN, USA), according to the manufacturer’s recommendation. Samples were labeled with Hy3, and the common reference (pool of all samples) was labeled with Hy5, by use of miRCURY LNA microRNA Array Power labeling kit (208032-A, Exiqon) and hybridized for 16 h. Slides were washed (208021, Exiqon), scanned on an Agilent (G2565CA) Microarray scanner and analyzed by the Genepix 6.0 software. Normalization and background correction was performed with “R” software using the “vsn” package (Bioconductor, open source software), and quadruplicate spots were averaged. Differential expression was assayed using the “limma” package (Bioconductor) by fitting the eBayes linear model and contrasting individual treatments with untreated controls. Log_2_ fold changes were calculated using the toptable function of the limma package.

### 4.8. mRNA and miRNA Analysis

Relative qRT-PCR of mRNA and miRNA were performed, as previously described [37,38]. Briefly, total RNA was extracted using the TriReagent protocol (Molecular Research Center, Inc., Cincinnati, Ohio), and RNA purity, integrity and quantity were examined by nanodrop (Nanodrop^®^ Technologies, Wilmington, DE, USA) and Bioanalyzer (Agilent 2100, Santa Clara, California) measurements. Relative quantitative mRNA PCR was performed on reverse transcribed cDNA (High Capacity cDNA RT kit; Applied Biosystems, Foster City, CA, USA). Primer sequences, amplification efficiencies (AE) and standard errors (SE): *BNP* forward: 5′-GATGCAGAAGCTGGAGA-3′, reverse: 5′-TCTGCTGGACCCGGAGGGTG-3′, AE: 2.046 and SE: 0.011; *Fibronectin* forward: 5′-CAGGGGTCACGTACCTCTTCAAAG-3′, reverse: 5′-CGAGGTGGAGTCCAAGTTACCAGA-3′, AE: 1.995 and SE: 0.010; *Procollagen-I* forward: 5′-ATCGTGGCTTCTCTGGTCTCCAG-3′, reverse: 5′-CAGGGAGACCGTTGAGTCCATCT-3′, AE: 2.039, SE: 0.004; *GAPDH* forward: 5′-GTCGGTGTGAACGGATTTGGC-3′, reverse: 5′-TGAAGGGGTCGTTGATGGCA-3′, AE: 2.079, SE: 0.008; *RPL13A* forward: 5′-GAAAGCGGATGAACACCAACCC-3′, reverse: 5′-GGGATCCCATCCAACACCTTGA-3′, AE: 1.958, SE: 0.003.

For miRNA qRT-PCR primers specific for rat and human miR-21 (#000397), miR-132 (#000457), miR-212 (#002551), miR-17 (#002308), miR-103 (#000439), miR-191 (#002299) and let-7f (#000382) were purchased from Applied Biosystems. Amplification and detection were performed using 7900HT Fast Real-Time PCR System (Applied Biosystems, Foster City, CA, USA). As recommended by others [39,40] and previously described [37,38], we used the qBase^+^ software to normalize all qRT-PCR data against multiple stably expressed control genes (information applied in respective figure legends).

### 4.9. Statistical Analysis

Results are represented as the mean ± SD. All analyses comprised independent experiments and two-way ANOVA, and two-tailed student’s *t*-tests were performed, as indicated [GraphPad Prism (5.0 version) software, La Jolla, CA, USA] to test significant levels. In the animal experiments, data on the effect of AngII and ET-1 were obtained from 6–8 and 3–5 animals, respectively. Data not following Gaussian distribution, as tested by D’Agostino’s normality test, were subjected to a Log_10_ transformation before statistical analysis and normality evidenced to follow Gaussian distribution prior to significance tests. Differences were considered to be significant at *p* < 0.05.

## 5. Conclusions

In conclusion, we found that miR-132 and -212 are increased in AngII-induced hypertension *in vivo*, in organs associated with blood pressure control, which partly mimics the “five miRNA” expression signature obtained by AT_1_R overexpression [19]. Cleary, the *in vivo* model adds to our understanding, as we can narrow down the miRNA changes after AngII, to miR-132 and -212. Importantly, our results show that functional inhibition of the AT_1_R reversed the miR-132 and -212 expression levels, demonstrating that AngII is responsible for the regulation, possibly via the Gαq-dependent pathway.

## Supplementary Information



## Figures and Tables

**Table 1:** miRNA microarray analysis showing significantly altered miRNA expression in the left ventricles from rats infused with AngII for 10 days compared to controls. Data is presented as log_2_ fold expression (*n =* 6–7) and sorted by *p*-value.

Microarray: miRNAs regulated by AngII		

Name of miRNA	Log fold change	*p*-value
		***p < 0.001
**21**	0.7016	1.89 × 10^−5^
**132**	0.1261	5.90 × 10^−5^
**105**	0.1883	9.28 × 10^−5^
**155**	0.1425	0.00012
**221**	0.3414	0.00052
**223**	0.4459	0.00085
**208b**	0.5560	0.00089
		**p < 0.01
**222**	0.1681	0.0022
**147b**	0.1151	0.0032
**26b**	−0.1153	0.0034
**15b**	0.2580	0.0057
**613**	−0.1047	0.0065
31*	0.1054	0.0075
**520b**	0.0938	0.0082
30c-1*	−0.1409	0.0084
**18b**	0.1334	0.0092
		*p < 0.05
**301a**	0.1537	0.010
**143**	0.1242	0.011
**434-5p**	0.1692	0.012
**484**	0.0855	0.014
**155**	0.1135	0.014
**379**	0.0813	0.015
**29c**	−0.2193	0.017
**936**	0.1682	0.018
**199a-5p**	0.1523	0.021
**201**	−0.1204	0.021
**101**	−0.1860	0.021
363*	0.2352	0.021
**760**	−0.0944	0.022
**944**	0.1056	0.023
200b*	0.1079	0.024
**30b**	−0.0888	0.025
**322**	−0.1890	0.026
**337-3p**	0.1065	0.026
29c*	−0.1247	0.028
302c*	0.1453	0.030
**193a-3p**	0.1665	0.030
**888**	−0.0852	0.033
**299-3p**	0.2128	0.035
**142-3p**	0.3121	0.035
517*	0.0746	0.041
**31**	0.3286	0.042
**194**	−0.0860	0.042
**701**	0.0671	0.042
**545**	−0.0834	0.043
**609**	0.0617	0.044
141*	−0.0928	0.045
**211**	0.0588	0.047
**373**	0.0712	0.048
218-1*	−0.0678	0.049

* means the passenger strand of the miRNA-miRNA* duplex; Passenger miRNA, may also have constitute a bioactive miRNA itself.
