# Supplementary material for: Angiotensin II Regulates microRNA-132/-212 in Hypertensive Rats and Humans
Source: Int J Mol Sci. 2013 May 27;14(6):11190–207. doi: 10.3390/ijms140611190 (PMC3709727; doi:10.3390/ijms140611190)
Supplement: Supplementary file 1 [file ijms-14-11190-s001.pdf]

Supplementary Information

Figure S1. qRT-PCR identification of miR-21 in the left ventricle of rats effected with AngII for 10 days. Statistical significance was tested by one-way ANOVA, ** $p < 0.005$. Values are shown as the mean ± SD; n = 5–7. Reference genes used for normalization; miR-17 and miR-191 (M: 0.356, CV: 0.123).

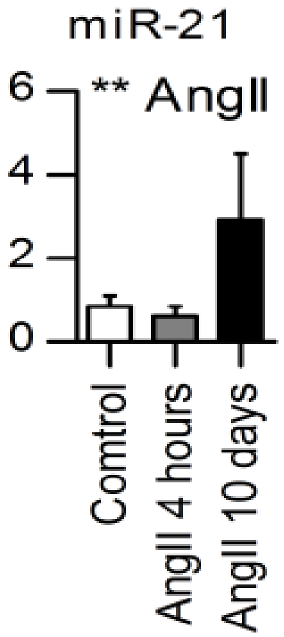

© 2013 by the authors; licensee MDPI, Basel, Switzerland. This article is an open access article distributed under the terms and conditions of the Creative Commons Attribution license (http://creativecommons.org/licenses/by/3.0/).
